# Northward shift of the Kuroshio Extension during 1993–2021

**DOI:** 10.1038/s41598-023-43009-w

**Published:** 2023-09-27

**Authors:** Yuma Kawakami, Hideyuki Nakano, L. Shogo Urakawa, Takahiro Toyoda, Kunihiro Aoki, Norihisa Usui

**Affiliations:** Department of Atmosphere, Ocean, and Earth System Modeling Research, Meteorological Research Institute, Tsukuba, Ibaraki Japan

**Keywords:** Climate sciences, Ocean sciences

## Abstract

The Kuroshio Extension (KE) flows eastward at the northern boundary of the North Pacific subtropical gyre. By transporting large amounts of seawater with heat, the KE contributes significantly to the formation of sea surface temperature (SST) fields. Recently, poleward shifts of major ocean gyres in the world ocean, including the North Pacific subtropical gyre, have been highlighted based on basin-scale changes in SST and sea surface height (SSH) distributions. However, a detailed investigation of the long-term meridional KE movement has not been presented. Investigation of KE path changes helps provide insights into long-term changes in the physical fields in the western North Pacific. In this study, we identified the KE path from satellite-derived SSH and surface current velocity data using a front identification method and showed that the KE migrated northward by approximately 210 km during 1993–2021. We further explored the cause of the northward KE shift based on atmospheric reanalysis data and numerical experiments using a high-resolution ocean general circulation model. It was revealed that the northward KE shift is mostly caused by the trend of wind stress curl in the North Pacific during 1993–2021.

## Introduction

The Kuroshio, the western boundary current of the North Pacific subtropical gyre, transports large amounts of seawater with heat from low- to mid-latitude North Pacific, contributing to heat distributions in the North Pacific. At the south of Japan, the Kuroshio goes eastward/northeastward with net volume transport of approximately 30–35 Sv (1 Sv = 10^6^ m^3^ s^−1^)^1–3^ in either the large meander path, nearshore non-large meander path, or offshore non-large meander path^4^ (Fig. S1). The Kuroshio separates from the Japan coast off the Boso Peninsula (approximately 35°N, 141°E) after passing through the meridionally oriented Izu–Ogasawara Ridge at around 140°E (Fig. S1), becoming an eastward jet known as the Kuroshio Extension (KE).

The KE flows eastward at the northern boundary of the North Pacific subtropical gyre. As the KE is accompanied by a large horizontal gradient of sea surface temperature (SST) on the northern side^5^, its meridional movement causes large SST changes. Many authors have demonstrated the meridional movement of the KE on interannual to decadal timescales and its significant influence on the SST field and the overlying atmosphere^5–8^.

It is well established that the KE fluctuates between dynamically stable and unstable states on a decadal timescale^9,10^. During the stable (unstable) state with steady (convoluted) paths, the KE tends to be stronger (weaker) and migrate northward (southward)^11^. Furthermore, during the unstable state, anti-cyclonic (cyclonic) mesoscale eddies are frequently detached from the KE to the north (to the south)^9,12^. Previous studies have suggested that the decadal KE variability is controlled by wind stress curl (WSC) over the North Pacific through westward propagating oceanic Rossby waves^9,10^ and by the Kuroshio path in the upstream^13,14^. The decadal KE variability and its associated eddy activities influence SST fields^5,15,16^, air–sea heat exchanges^14,15,17^, and water mass formations^18–21^ in the western North Pacific.

Despite many studies on decadal KE variability, our understanding of the long-term meridional KE path change remains limited. Previously, Yang et al.^22^ reported that major ocean gyres in the world ocean, including the North Pacific subtropical gyre, have shifted poleward in the past few decades because of a poleward shift of large-scale atmospheric circulations^23,24^. However, discussions focusing on the KE were not necessarily presented, as such a long-term meridional KE movement has not been sufficiently understood yet.

The purpose of this study is to investigate the long-term meridional KE position change and to understand its cause. Satellite measurements have accumulated sea surface height (SSH) and surface current velocity data for almost three decades since 1993. Nakano et al.^25^ developed an elaborate method for identifying the KE axis (and other strong current axes in the North Pacific) from SSH and surface current velocity fields. These data and the method are useful for investigating the KE path. We analyzed the latitudinal position of the KE from satellite-derived SSH and surface current velocity data using the method of Nakano et al.^25^ and explored its temporal change during 1993–2021. The previous work suggested that the poleward shift of the ocean gyres is attributable to changes in wind stress forcing^22^. We also investigated a long-term change in wind stress forcing over the North Pacific by analyzing atmospheric reanalysis data and evaluated its impact on the KE path through numerical experiments using a high-resolution ocean general circulation model.

## Results

### Northward migration of the KE

**Figure 1:**
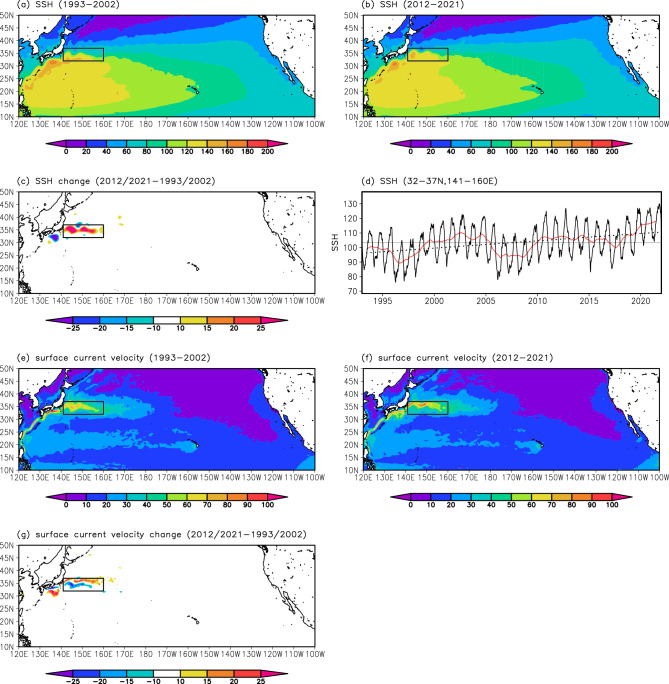
Long-term changes in SSH and surface current velocity fields in the North Pacific from satellite observations. Mean SSH (cm) distributions for (**a**) 1993–2002 and (**b**) 2012–2021, and (**c**) their difference. The black rectangle indicates the KE region (32–37°N, 141–160°E). (**d**) Timeseries of SSH (cm) averaged in the KE region. The black solid line indicates daily SSH values. The red line represents the 365-day running mean. The gray horizontal bar indicates the average value for 1993–2021. The linear trend for 1993–2021 is also indicated by a dotted line. Global mean sea-level rise due to expansion/contraction and freshwater fluxes (3.58 mm year^−1^ based on tide gauge observations and satellite altimetry measurements during 1993–2015^48^) was removed before drawing the panels (**a**)–(**d**). (**e**)–(**g**) Same as (**a**)–(**c**), but for surface current velocity (cm s^−1^). All plots were generated with GrADS v2.0.2 (http://cola.gmu.edu/grads/grads.php).

We first compared observed SSH fields in the western North Pacific between the two 10-year periods of 1993–2002 and 2012–2021 (i.e., the first and recent 10 years of satellite-derived SSH product) (Fig. 1a and b) to understand long-term KE path changes. The result indicated that SSH markedly increased in the KE region from 1993–2002 to 2012–2021 (Fig. 1c). The timeseries of mean SSH in the KE region (32–37°N, 141–160°E) further revealed that the SSH has increased since 1993 with decadal-scale variations (Fig. 1d). Surface current velocity fields in 1993–2002 and 2012–2021 were also compared (Fig. 1e and f). Their difference showed a meridional dipole pattern in the KE region (Fig. 1g): positive anomalies representing surface current acceleration were detected zonally at approximately 35–37°N and negative anomalies reflecting surface current deceleration were found toward the south (at approximately 33–35°N). These SSH and surface current velocity changes suggest that the mean latitudinal position of the KE in 2012–2021 was further north compared with that in 1993–2002, implying that the KE has migrated northward since 1993.

**Figure 2:**
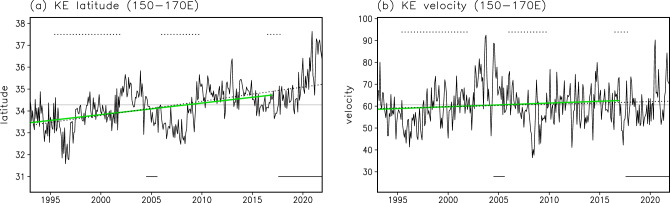
Long-term changes in the KE latitude and KE velocity from satellite observations. Timeseries of (**a**) the KE latitude (°N) and (**b**) the KE velocity (cm s^−1^). The solid and dotted lines indicate monthly values and the linear trend for 1993–2021, respectively. The gray line indicates the mean value for 1993–2021. The green line indicates the linear trend for 1993–2016. The solid horizontal bars mean periods of the Kuroshio large meander. The dotted horizontal bars indicate periods when the KE is in the unstable state. All plots were generated with GrADS v2.0.2 (http://cola.gmu.edu/grads/grads.php).

**Figure 3:**
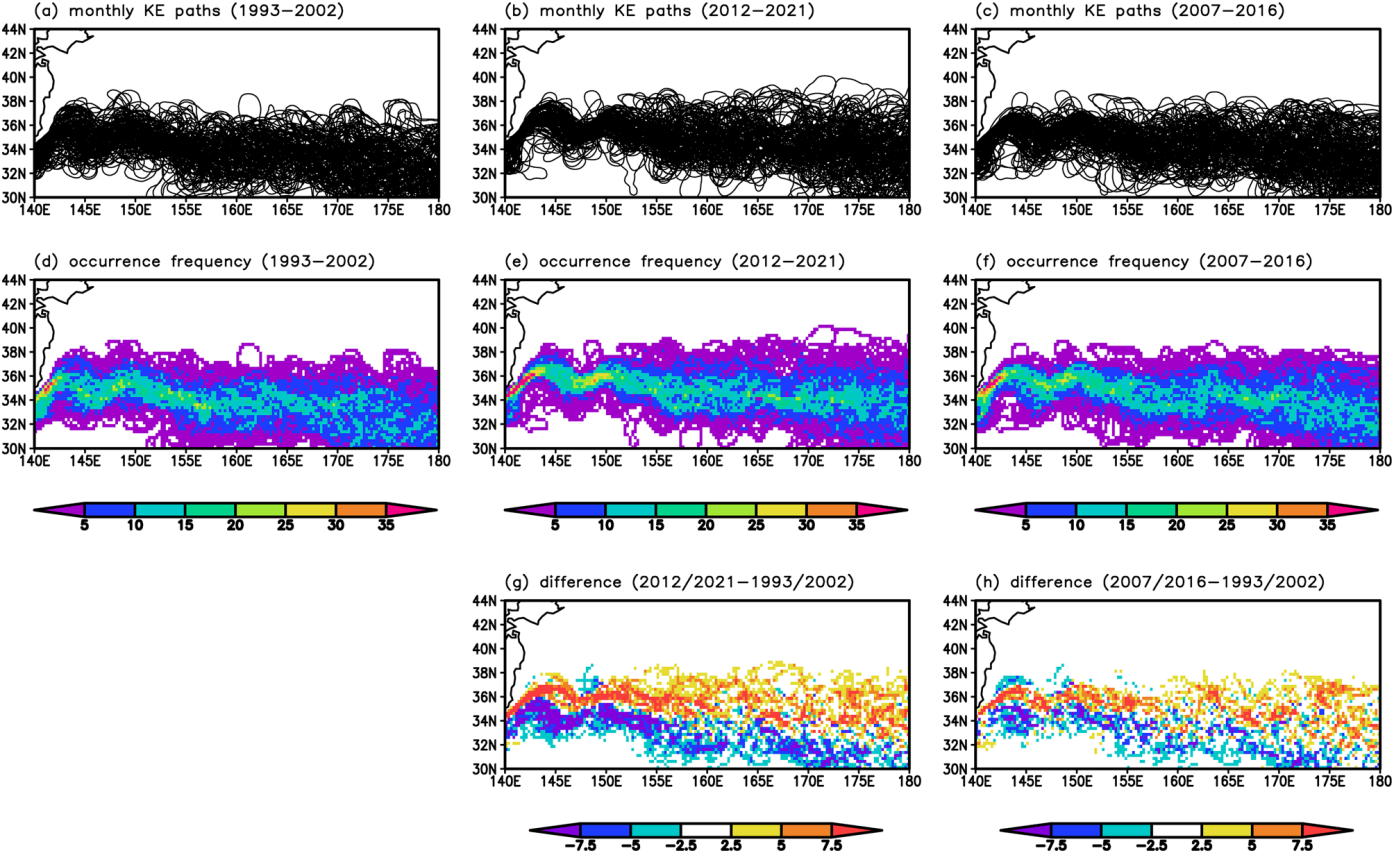
Long-term changes in the KE-axis location from satellite observations. Spaghetti diagrams of monthly KE axes for (**a**) 1993–2002, (**b**) 2012–2021, and (**c**) 2007–2016. (**d**)–(**f**) Occurrence frequency (%) of the KE axis for 1993–2002, 2012–2021, and 2007–2016. (**g**) Difference of occurrence frequency (%) of the KE axis between 2012–2021 and 1993–2002. (**h**) Same as (**g**), but for the difference between 2007–2016 and 1993–2002. All plots were generated with GrADS v2.0.2 (http://cola.gmu.edu/grads/grads.php).

Next, to investigate the meridional KE movement in more detail, we analyzed the latitudinal position of the KE during 1993–2021 focusing on the KE axis. We identified the KE axis using a front identification method developed by Nakano et al.^25^ and defined the KE latitude based on the zonally averaged latitudinal position of the KE axis (see “Methods”). As a result, the KE latitude showed a positive trend (Fig. 2a): the KE migrated northward by approximately 210 km from 1993 to 2021 (7.20 km year^−1^, exceeding the significance level of 0.05) with decadal-scale fluctuations. To visually confirm the northward KE shift, we further plotted spaghetti diagrams and occurrence frequency distributions of the KE axis for the periods of 1993–2002 and 2012–2021 (Fig. 3a,b,d,e,g). The results showed that the KE was located at a higher latitude in 2012–2021 than in 1993–2002.

The KE path is considered to be related to the Kuroshio path south of Japan. Previous studies have suggested that the occurrence of the Kuroshio large meander causes the KE to be in a stable state with a northerly path^13,14^. After 1993, there were two Kuroshio large meander events: one occurred from July 2004 to August 2005^26–28^, and the other has been ongoing since August 2017, at the time of writing this manuscript^14,29–31^. It is pointed out that the obtained northward KE shift may strongly reflect the recent Kuroshio large meander. To remove the influence of the latest Kuroshio large meander, we further investigated the KE path changes during 1993–2016. As a result, we obtained a statistically significant positive trend of the KE latitude (5.89 km year^−1^, exceeding the significance level of 0.05) (Fig. 2a). Furthermore, a comparison of the KE axis occurrence frequency between the two 10-year periods of 2007–2016 and 1993–2002 (Fig. 3a,c,d,f,h) showed that the KE was located at a higher latitude in 2007–2016 than in 1993–2002. These results suggest that the northward KE shift is a long-term change rather than a signal caused by the recent Kuroshio large meander event. On the other hand, a close examination of Fig. 2a reveals that the KE latitude in recent years has been unusually high compared with previous years. This anomalous situation may be related to the Kuroshio large meander path since August 2017. The influence of the Kuroshio large meander on the KE is an interesting issue that will require further investigation in the future.

We also investigated a long-term change in the KE velocity. A comparison of mean surface current velocity fields between the two periods of 1993–2002 and 2012–2021 reveals that the maximum value in the KE region is slightly higher in 2012–2021 than in 1993–2002 (Fig. 1e and f). This may reflect an intensification of the KE during 1993–2021. However, no significant trend was obtained for the KE velocity (Fig. 2b): although the trend of the KE velocity is positive (0.12 cm s^−1^ year^−1^, non-significant), its associated KE velocity change is much smaller than the amplitude of interannual to decadal-scale variations.

### Changes in wind stress forcing in the North Pacific

**Figure 4:**
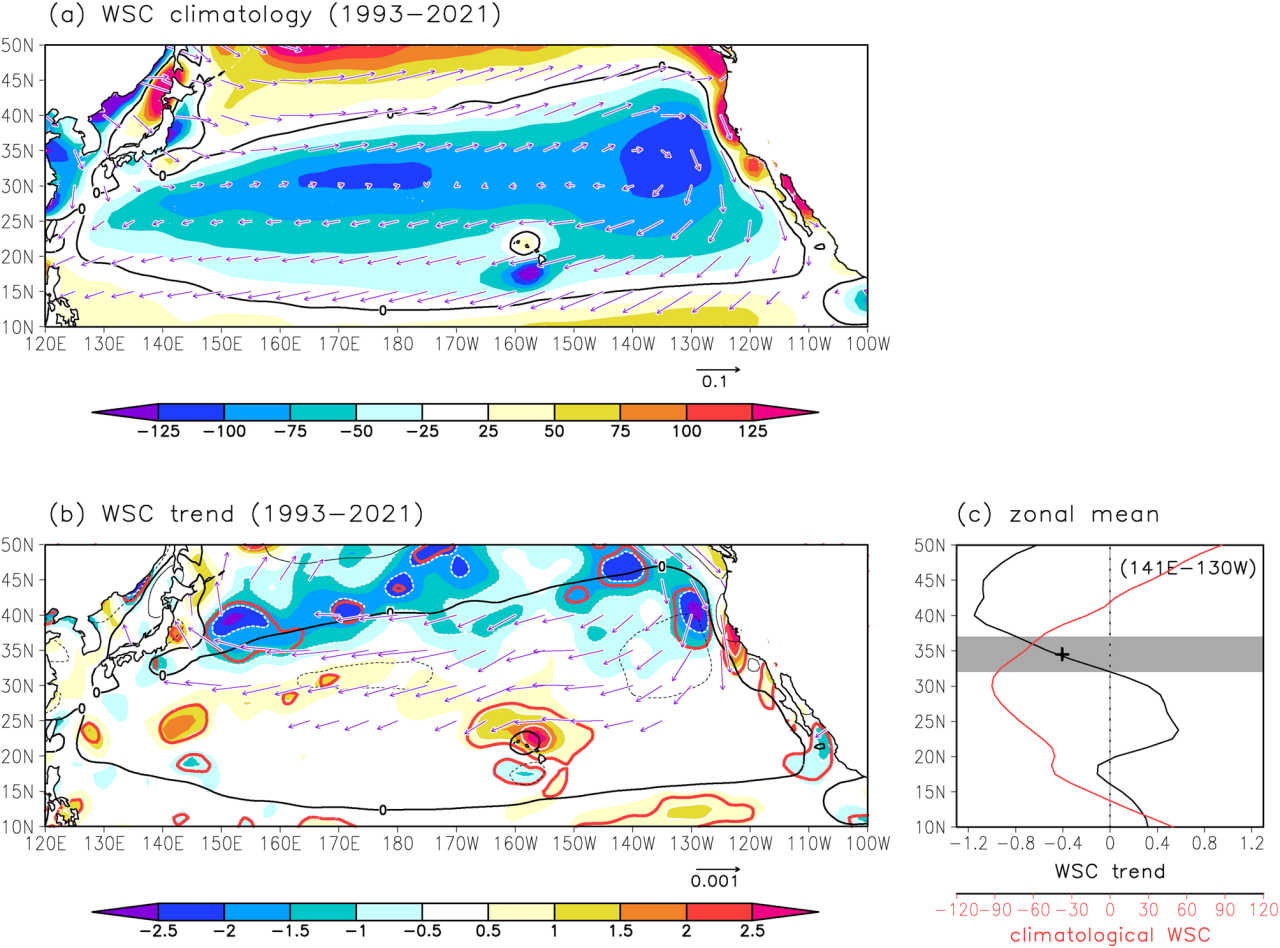
Long-term WSC changes over the North Pacific from atmospheric reanalysis data. (**a**) Climatology of WSC (10^−9^ N m^−3^) (average for 1993–2021). Vectors indicate surface wind stress climatology (N m^−2^). (**b**) Linear trends of WSC (10^−9^ N m^−3^ year^−1^). The red lines indicate statistically significant values. Contour denotes WSC climatology with intervals of 100 × 10^−9^ N m^−3^. Vector indicates linear trends of surface wind stress (N m^−2^ year^−1^) in the North Pacific. Vectors whose magnitude is smaller than 0.0005 N m^−2^ year^−1^ are not shown. (**c**) Meridional distributions of zonal mean WSC trends (10^−9^ N m^−3^ year^−1^)(black) and WSC climatology (10^−9^ N m^−3^) (red) between 141°E–130°W. Gray shading is drawn for the meridional range of 32–37°N, where the KE is located. The cross mark indicates the average value of the WSC trend for 32–37°N, 141°E–130°W. All plots were generated with GrADS v2.0.2 (http://cola.gmu.edu/grads/grads.php).

As the KE is a part of the wind-driven circulation in the North Pacific subtropical gyre, the meridional movement of the KE is attributable to changes in wind stress forcing. Next, we investigated a long-term change in the WSC field over the North Pacific based on atmospheric reanalysis data. In the subtropical North Pacific between the westerlies and trade winds, WSC is negative overall (Fig. 4a). During 1993–2021, WSC exhibited negative trends around the zero contours of WSC climatology (defined as the average for 1993–2021) in the mid- to high-latitudes (Fig. 4b). This WSC trend reflects weakening of the westerlies at approximately 35°N and/or northward shift of atmospheric circulations (i.e., westerlies and trade winds), which are consistent with a previous study^23^. The zonal mean values of the WSC trend were negative in the north of 32°N (Fig. 4c). Note that the KE is located north of 32°N. Here, the negative trend of WSC around its climatological zero contours means that regions with negative WSC values have extended northward. Considering that the North Pacific subtropical gyre originated from negative WSC, the obtained WSC trend implies the northward expansion of the North Pacific subtropical gyre and is consistent with the northward shift of the KE.

### Influences of wind stress forcing change on the KE path

Analyses of the atmospheric reanalysis data implied that the northward KE shift is associated with the long-term WSC trend in the North Pacific. However, the impacts of the WSC trend on the KE were not sufficiently confirmed. Ocean models are a useful tool for investigating oceanic responses to changes in atmospheric forcings. To evaluate the influence of the WSC trend on the KE, we performed numerical simulations with the high-resolution (grid spacing ~ 10 km) North Pacific model (NP model)^32,33^ developed at the Meteorological Research Institute (MRI). We conducted two model experiments by imposing different atmospheric conditions (see “Methods”); in the first experiment (CTRL run), the NP model was driven by raw 3-hourly atmospheric forcing data; the second experiment (DTND run) is identical to the CTRL run except that we imposed the detrended wind stress forcing after 1993. Here, previous studies showed that on a decadal timescale, the KE responds to WSC changes in the central North Pacific with a lag of few years^9–11^. In that case, the time lag should probably be considered in the detrend of wind stress forcing. However, on the long-term KE path change, the forcing region and response time have not been necessarily clarified. Therefore, in this study we investigated impacts of WSC trends after 1993 over the North Pacific including no lag time for simplicity.

**Figure 5:**
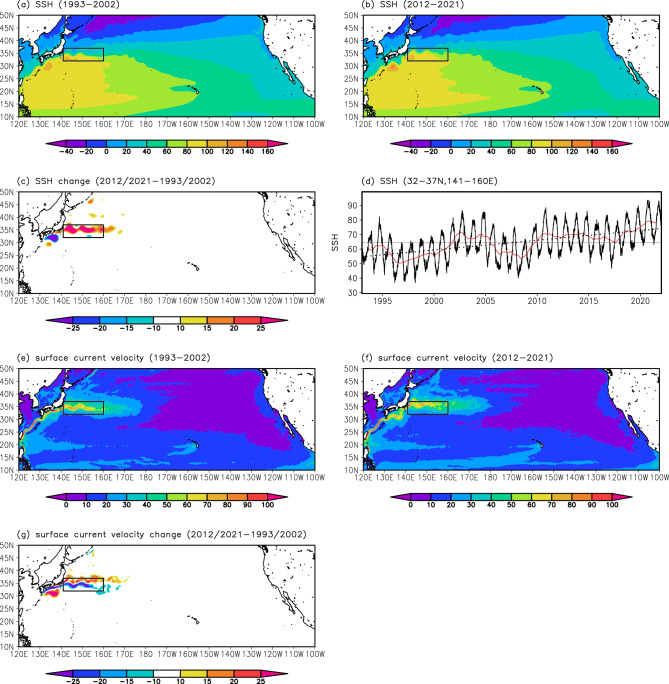
Long-term changes in SSH and surface current velocity fields in the North Pacific from the CTRL run. Same as Fig. 1, but from the CTRL run. Signals of sea-level rise due to expansion/contraction and freshwater fluxes were not included because the water volume was conserved in the simulation using the Boussinesq approximation and virtual salinity flux was adopted instead of surface freshwater flux^39^. All plots were generated with GrADS v2.0.2 (http://cola.gmu.edu/grads/grads.php).

**Figure 6:**
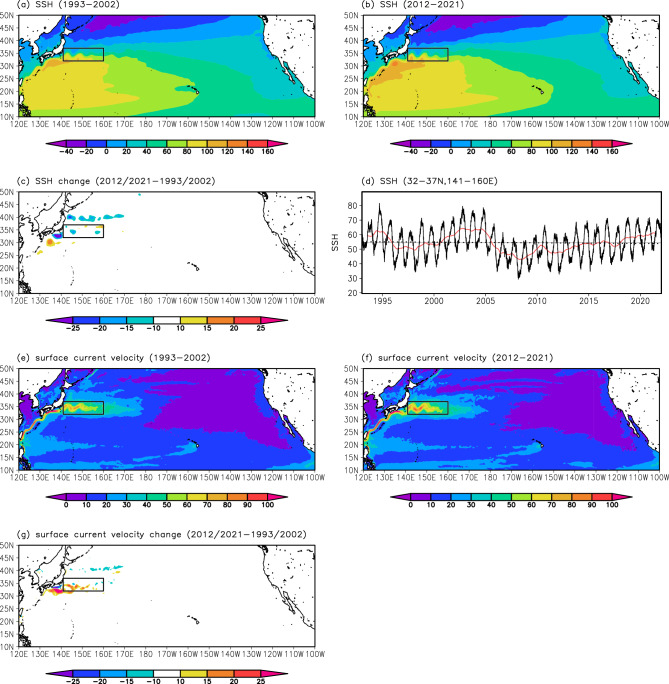
Long-term changes in SSH and surface current velocity fields in the North Pacific from the DTND run. Same as Fig. 5, but from the DTND run. All plots were generated with GrADS v2.0.2 (http://cola.gmu.edu/grads/grads.php).

The CTRL run reproduced observed SSH changes during 1993–2021 (Figs. 1a–d, 5a–d). The simulated SSH in the KE region increased between 1993 and 2021 with decadal-scale variations (Fig. 5d), consistent with the observation result (Fig. 1d). Changes in the surface current velocity field were also reproduced in the CTRL run (Figs. 1e–g, 5e–g): acceleration and deceleration signals were found in the northern and southern parts of the KE region, respectively, similar to observations. Note that the wind stress forcing in the CTRL run well represented the linear trends for 1993–2021 found in the atmospheric reanalysis data (Fig. S2; see also Fig. 4). On the other hand, in the DTND run, the observed SSH and surface current velocity changes were not reproduced (Fig. 6).

**Figure 7:**
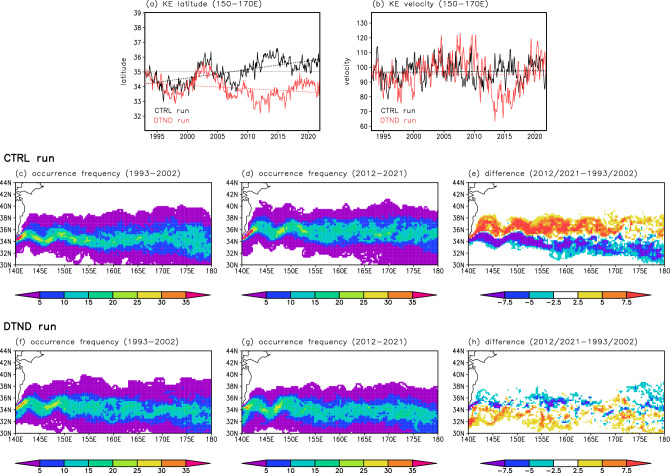
Long-term changes in the KE from the NP model simulations. (**a**) and (**b**) Same as Fig. 2a and b, but from the NP model simulations. The black and red lines indicate the CTRL and DTND runs, respectively. The gray line indicates the mean value in the CTRL run. (**c**)–(**e**) Same as Fig. 3d,e,g, but from the CTRL run. (**f**)–(**h**) Same as Fig. 3d,e,g, but from the DTND run. All plots were generated with GrADS v2.0.2 (http://cola.gmu.edu/grads/grads.php).

The KE latitudes were also calculated from the NP model simulations (Fig. 7a). In the CTRL run, the KE latitude increased between 1993 and 2021 with a decadal-scale variation, consistent with satellite observations (Fig. 2a). The northward shift from 1993 to 2021 was approximately 190 km (6.53 km year^−1^, exceeding the significance level of 0.05). A comparison of the KE axis occurrence frequency in 2012–2021 and 1993–2002 also indicated the northward shift of the KE in the CTRL run (Fig. 7c–e). In contrast, in the DTND run, the KE latitude decreased during 1993–2021 with a rate of 2.53 km year^−1^ (exceeding the significance level of 0.05) (Fig. 7a). The KE axis occurrence frequency in 1993–2002 and 2012–2021 also showed that the KE migrated southward in the DTND run (Fig. 7f–h). These simulation results suggest that the observed northward shift of the KE in recent decades is attributable to changes in wind stress forcing in the North Pacific.

Furthermore, we investigated using the Sverdrup stream function, which represents a large-scale ocean surface current field expected from wind stress forcings. We computed mean Sverdrup stream functions for 1993–2002 and 2012–2021 and compared them to estimate the meridional KE shift due to changes in wind stress forcing (Fig. S3). As a result, a northward shift of approximately 1.0–1.5° (about 111–167 km) was obtained around the KE region in the CTRL run. This result supports the hypothesis that the northward shift of the KE can be explained by changes in wind stress forcing. On the other hand, in the DTND run, no significant change was found in the Sverdrup stream function, whereas the southward shift was analyzed (Fig. 7a). This point is discussed in the following section. Note that the KE is located much further south (around 35°N) than expected by the wind stress forcing (i.e., at the Sverdrup stream function = 0 at approximately 40–45°N) (Fig. S3). This feature is called “premature separation” and is common with other western boundary current extensions such as the separated Gulf Stream. Kubokawa^34^ investigated this issue using a two-layer quasi-geostrophic rectangle model and showed that the separation latitude (e.g., the KE latitude) depends on a meridional profile of wind stress forcings (i.e., the Sverdrup stream function) under realistic conditions. Our simulation results indicating the northward KE migration with the northward shift of the Sverdrup stream function are consistent with the results of Kubokawa^34^.

In addition, we investigated long-term changes in the KE velocity from model simulations. As a result, both the CTRL and DTND runs showed no significant trend in the KE velocity (Fig. 7b), consistent with observations (Fig. 2b). Here, the simulated KE velocity was larger than the observed KE velocity, reflecting the difference in horizontal resolutions between the NP model simulations and satellite measurements.

## Summary and discussions

Recently, poleward shifts of major ocean gyres, including the North Pacific subtropical gyre, have been highlighted^22^. This implies that the KE at the northern boundary of the North Pacific subtropical gyre has also changed its latitudinal position northward. However, a detailed investigation of KE path changes has not been presented. In this study, based on satellite observations and the front identification method, we demonstrated that the KE migrated northward by approximately 210 km between 1993 and 2021. The cause of the KE shift was also investigated using atmospheric reanalysis data and high-resolution ocean general circulation model simulations. The results revealed that the WSC trends in the North Pacific during 1993–2021 were responsible for the northward KE shift.

**Figure 8:**
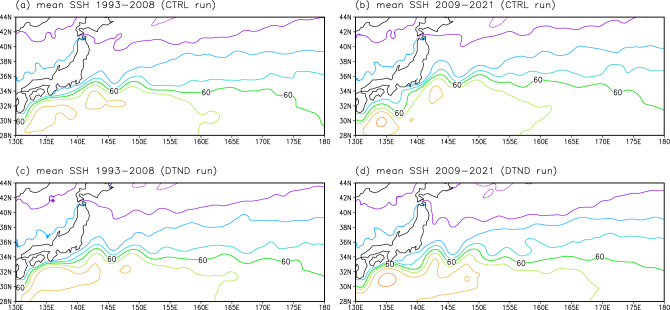
Mean SSH field from NP model simulations. SSH (cm; contour intervals of 20 cm) from the CTRL run averaged for (**a**) 1993–2008 and (**b**) 2009–2021. (**c**) and (**d**) Same as (**a**) and (**b**), but from the DTND run. All plots were generated with GrADS v2.0.2 (http://cola.gmu.edu/grads/grads.php).

Notably, the KE in the DTND run migrated southward during 1993–2021 (Fig. 7a) despite remaining at the same latitudes is expected from the wind stress forcing (Fig. S3). A close examination of Fig. 7a reveals that the KE latitude in the CTRL and DTND runs fluctuated similarly before 2008 except for a long-term trend, but behaved differently after 2008: the KE latitude in the two experiments separated suddenly after 2008 due to an abrupt southward movement of the KE in the DTND run. We also found that the mean Kuroshio path south of Japan, the upstream of the KE, was similar in the two experiments before 2008 but differed greatly after 2008 (Fig. 8); in the CTRL and DTND runs for 1993–2008, the mean Kuroshio path appears to be the nearshore non-large meander path (Fig. 8a and c); after 2008, the mean Kuroshio path in the CTRL run appears to be the large meander path (Fig. 8b), whereas that in the DTND run appears to be the offshore non-large meander path (Fig. 8d). The nearshore non-large meander path and large meander path passing the northern part of the Izu–Ogasawara Ridge (Fig. S1) tend to result in the stable and northerly KE path^13,14^. On the other hand, the offshore non-large meander path passing the southern part of the Izu–Ogasawara Ridge (Fig. S1) tends to result in the unstable and southerly KE path^13,35^. Therefore, the difference in the KE latitude between CTRL and DTND runs suddenly occurred in 2008 is thought to reflect the different Kuroshio path changes between the two runs. The Kuroshio path is influenced by WSC changes over the North Pacific^14,35^. Usui et al.^36^ suggested that an oceanic condition with small net Kuroshio transport reflecting positive WSC anomalies in the North Pacific subtropical gyre is favorable to the occurrence and persistence of the Kuroshio large meander. In the CTRL run, WSC showed positive trends in the North Pacific subtropical gyre (Fig. S2), consistent with Kuroshio path changes (Fig. 8a and b). On the other hand, in the DTND run, such WSC trends are removed (see “Methods”). Wind stress forcing in the DTND run with no linear trend would cause the offshore non-large meander Kuroshio path after 2008, resulting in the southerly path of the KE.

As the KE is accompanied by the SST front on the northern side^5^, its northward shift causes a large SST increase in the surrounding oceans. A previous observational study^37^ discovered rapid sea surface warming in the KE region and suggested the impact of KE path changes. The northward shift of the KE and its associated enhanced warming may influence regional fisheries by changing marine resource distributions and the overlying atmosphere via air–sea interactions. Monitoring the KE path change and understanding its oceanic and atmospheric impacts would aid in climate change adaptation.

## Methods

### Observational data

We used satellite-derived daily SSH and surface current velocity data from the Copernicus Marine Environment Monitoring Service. The horizontal resolution of these data is 0.25° (longitude) × 0.25° (latitude). These SSH and surface current velocity data date back to 1993. We analyzed the delayed time product for 1993–2021.

We also used monthly surface wind stress data from Japanese 55-year Reanalysis (JRA-55)^38^. The horizontal resolution of these data is 1.25° (longitude) × 1.25° (latitude). The WSC was calculated over the North Pacific.

### Model experiments

We performed numerical simulations using the NP model^32,33^ developed by the MRI. This model is based on the MRI Community Ocean Model (MRI.COM)^39^, which is one of the standard ocean general circulation models used in the international intercomparison project (OMIP2)^40^. The MRI.COM solves primitive equations using Boussinesq and hydrostatic approximations and adopts a vertically re-scaled height coordinate system, where sea-level undulations are reflected throughout the water column^41^. The NP model domain is 15°S–63°N and 99°E–75°W. The NP model has a horizontal resolution of 1/11° (longitude) × 1/10° (latitude) with 60 vertical levels; the layer thickness increases with depth from 2 m at the top to 700 m for the lowest layer. The NP model is nested in a global ocean model with a horizontal resolution of 1° (longitude) × 1/2° (latitude) (GONDOLA_100)^42^ using a 1-way off-line nesting method. The basic performance of the NP model was presented in previous studies^33,43^.

The atmospheric forcings used in this study (surface shortwave and longwave radiation fluxes, zonal and meridional wind speed at 10-m height, sea-level pressure, precipitation, and air temperature and specific humidity at 10-m height) are from the 3-hourly JRA-55-do dataset^44^. Surface wind stress, latent and sensible heat fluxes, and evaporation are calculated using bulk formulas^45,46^. The initial conditions were created by GONDOLA_100 simulation with the JRA-55-do dataset from the World Ocean Atlas 2013 climatology^47^; we imposed the 60-year-long JRA-55-do forcing (1959–2018) repeatedly for five cycles (i.e., 300 years in total), and the restart of the 5th cycle at 00 UTC on January 1, 1960, was used for the initial conditions. The model experiments covered a 62-year period from 1960 to 2021 (CTRL run). In this study, we focus on the events that occurred in the 1993–2021 period when delayed time satellite-derived SSH and surface current velocity data are available.

To investigate the influences of wind stress forcing changes on the KE, we conducted another experiment in which different atmospheric conditions were imposed (DTND run). The DTND run was similar to the CTRL run, except that we imposed the detrended 3-hourly surface wind stress forcings after 1993: we constructed the detrended surface wind stress forcing data for the DTND run using 3-hourly outputs of the CTRL run and directly imposed these data.

### Identification of the KE axis

To investigate temporal changes in the KE, we focused on the KE axis. In this study, the KE axis was identified using the front identification method of Nakano et al.^25^: we detected the KE axis from each monthly SSH and surface current velocity field as a specific SSH contour, which is characterized by the maximum surface current velocity in the western North Pacific. See Nakano et al.^25^ for details of this method.

The method of considering a specific SSH contour as the KE axis has been extensively used^9,13^. The SSH value of the KE axis contour has been typically defined as time-independent. However, the use of the fixed SSH value for the KE axis identification may introduce uncertainty because the SSH value representing the KE axis may change over time. On the other hand, the method of Nakano et al.^25^ can identify the KE axis without such uncertainty because the SSH value of the KE-representing contour is determined in each month (i.e., as time-dependent) based on SSH and surface current velocity fields.

### Definition of the KE latitude and KE velocity

In this study, the KE latitude was defined as the mean latitudinal position of the KE axis between 150°E and 170°E. The KE velocity was defined as the mean velocity along the KE axis between 150°E and 170°E. We verified that the results were not affected by the choice of the zonal range to average; for instance, almost identical results were obtained if the KE latitude and KE velocity were defined in 145–165°E**.**

### Significance test of linear trends

In this study, we tested the statistical significance of linear trends using Student’s two-sided t-test, with a significance level of 0.05 for all trends.

### Supplementary Information

Supplementary Figures.

## Data Availability

All data are available from the corresponding author upon reasonable request. SSH and surface current velocity data can be obtained from the Copernicus Marine Environment Monitoring Service website (https://data.marine.copernicus.eu). JRA-55 can be downloaded from DIAS’s website (https://diasjp.net/en/).
