# Supplementary material for: Northward shift of the Kuroshio Extension during 1993–2021
Source: Sci Rep. 2023 Sep 27;13:16223. doi: 10.1038/s41598-023-43009-w (PMC10533485; doi:10.1038/s41598-023-43009-w)
Supplement: Supplementary file 1 — Supplementary Figures. [file 41598_2023_43009_MOESM1_ESM.pdf]

Supplementary information for

Northward shift of the Kuroshio Extension during 1993−2021

Yuma Kawakami[1,*], Hideyuki Nakano[1], L. Shogo Urakawa[1], Takahiro Toyoda[1], Kunihiro Aoki[1], and Norihisa Usui[1]

[1]Department of Atmosphere, Ocean, and Earth System Modeling Research, Meteorological Research Institute, Tsukuba, Ibaraki, Japan

Contents of this file:

Figures S1 to S3

[*]Corresponding author: Yuma Kawakami (y-kawakami@mri-jma.go.jp)

Department of Atmosphere, Ocean, and Earth System Modeling Research, Meteorological Research Institute, Tsukuba, Ibaraki, Japan

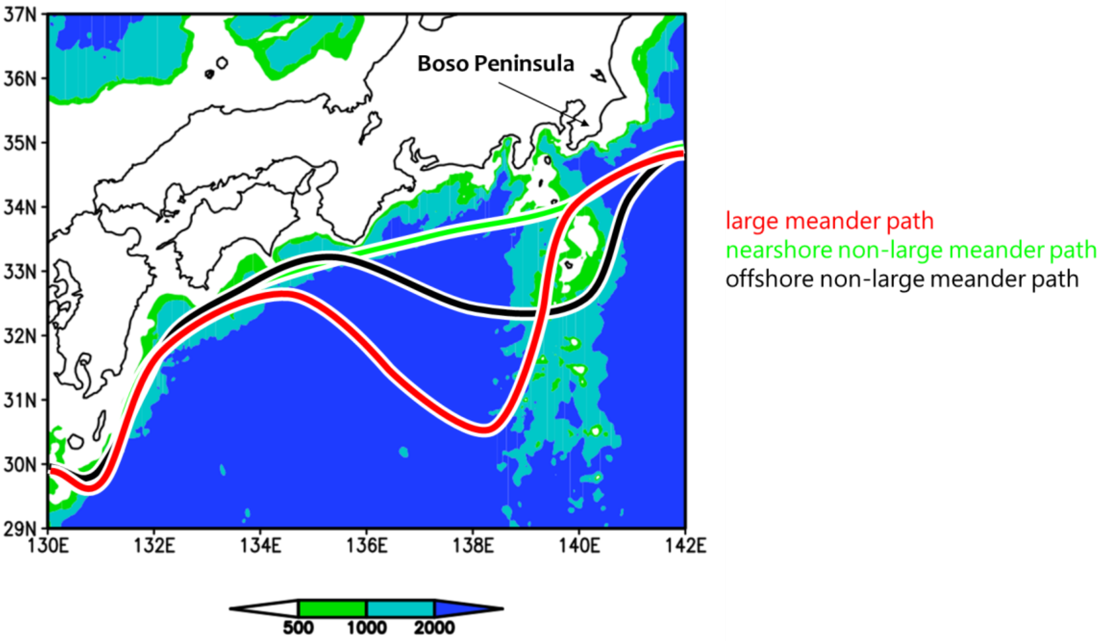

Figure S1

Typical Kuroshio paths south of Japan. The red, green, and black lines indicate schematic illustrations of the large meander path, nearshore non-large meander path, and offshore non-large meander path, respectively, based on Kawabe[4]. Color shading indicates bottom topography (m). The plot was generated with GrADS v2.0.2 (http://cola.gmu.edu/grads/grads.php).

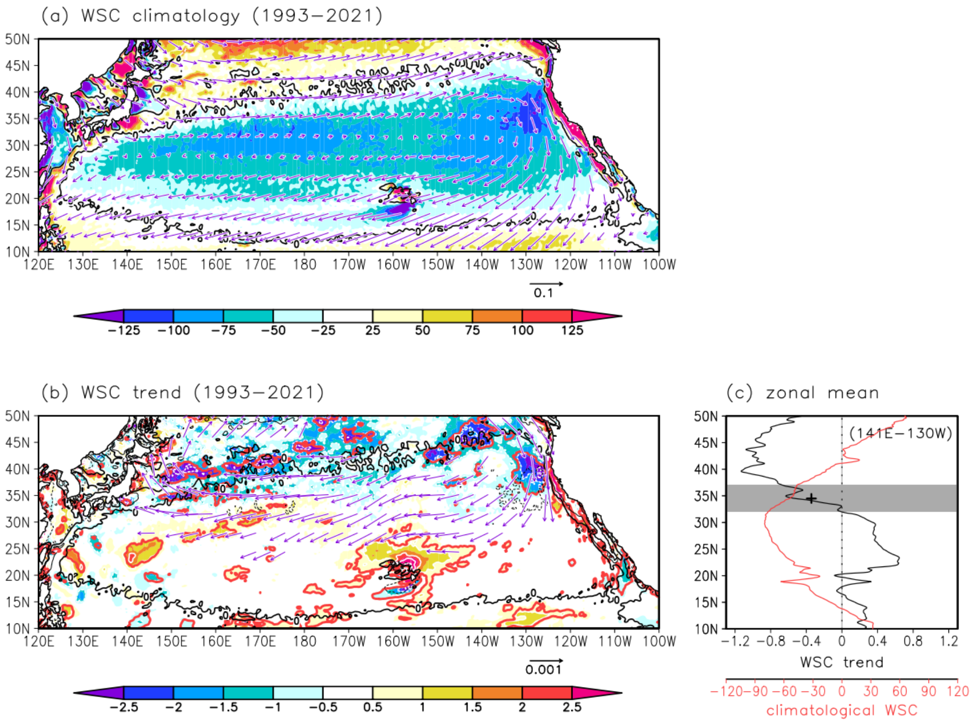

Figure S2

Long-term WSC changes over the North Pacific from the CTRL run. Same as Figure 4, but from the CTRL run. All plots were generated with GrADS v2.0.2 (http://cola.gmu.edu/grads/grads.php).

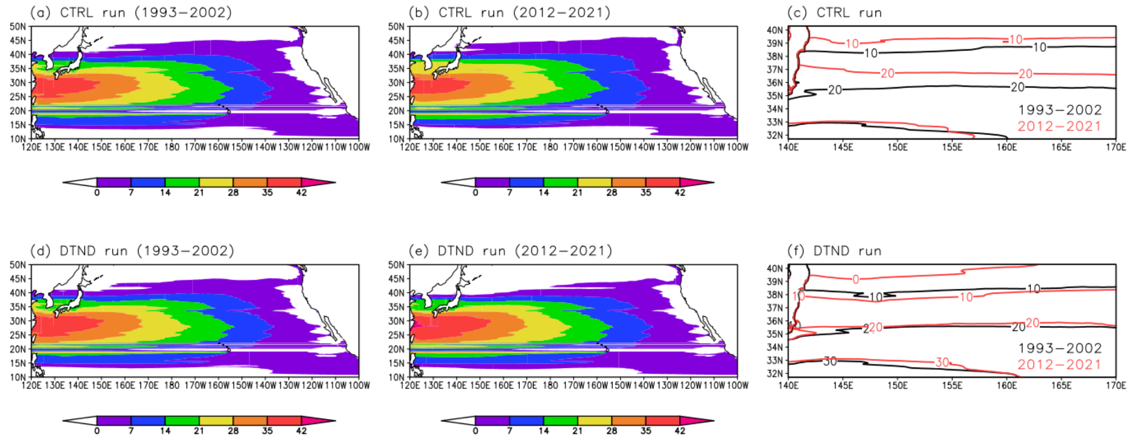

Figure S3

Long-term changes in Sverdrup stream function from NP model simulations. Sverdrup stream function (Sv) averaged for (**a**) 1993–2002 and (**b**) 2012–2021 from the CTRL run. (**c**) Enlarged figure of the KE region in panels (**a**) and (**b**). The black and red lines indicate the average for 1993–2002 and 2012–2021, respectively. (**d**)–(**f**) Same as (**a**)–(**c**), but from the DTND run. All plots were generated with GrADS v2.0.2 (http://cola.gmu.edu/grads/grads.php).
